# Key ethical issues encountered during COVID-19 research: a thematic analysis of perspectives from South African research ethics committees

**DOI:** 10.1186/s12910-023-00888-y

**Published:** 2023-02-15

**Authors:** Theresa Burgess, Stuart Rennie, Keymanthri Moodley

**Affiliations:** 1 Centre for Medical Ethics and Law, WHO Collaborating Centre for Bioethics, Faculty of Medicine and Health Sciences, Stellenbosch University, Cape Town, South Africa; 2 Division of Physiotherapy, Department of Health and Rehabilitation Sciences, Faculty of Health Sciences, University of Cape Town, F45 Old Main Building, Groote Schuur Hospital, Anzio Road, Observatory, Cape Town, 7725 South Africa; 3 Department of Social Medicine, UNC Bioethics Center, University of North Carolina, Chapel Hill, USA

**Keywords:** Research ethics committees, COVID-19, Pandemic, Public health emergency, Research ethics

## Abstract

**Background:**

The COVID-19 pandemic presents significant challenges to research ethics committees (RECs) in balancing urgency of review of COVID-19 research with careful consideration of risks and benefits. In the African context, RECs are further challenged by historical mistrust of research and potential impacts on COVID-19 related research participation, as well as the need to facilitate equitable access to effective treatments or vaccines for COVID-19. In South Africa, an absent National Health Research Ethics Council (NHREC) also left RECs without national guidance for a significant duration of the COVID-19 pandemic. We conducted a qualitative descriptive study that explored the perspectives and experiences of RECs regarding the ethical challenges of COVID-19 research in South Africa.

**Methods:**

We conducted in-depth interviews with 21 REC chairpersons or members from seven RECs at large academic health institutions across South Africa that were actively involved in the review of COVID-19 related research from January to April 2021. In-depth interviews were conducted remotely via Zoom. Interviews (60–125 min) were conducted in English using an in-depth interview guide, until data saturation was achieved. Audio-recordings were transcribed verbatim and field notes were converted into data documents. Line-by-line coding of transcripts was performed, and data were organised into themes and sub-themes. An inductive approach to thematic analysis was used to analyse data.

**Results:**

Five main themes were identified, namely: rapidly evolving research ethics landscape, extreme vulnerability of research participants, unique challenges to informed consent, challenges to community engagement during COVID-19, and overlapping research ethics and public health equity issues. Sub-themes were identified for each main theme.

**Conclusions:**

Numerous, significant ethical complexities and challenges were identified by South African REC members in the review of COVID-19 related research. While RECs are resilient and adaptable, reviewer and REC member fatigue were major concerns. The numerous ethical issues identified also highlight the need for research ethics teaching and training, especially in informed consent, as well as the urgent requirement for the development of national guidelines for research ethics during public health emergencies. Further, comparative analysis between different countries is needed to develop the discourse around African RECs and COVID-19 research ethics issues.

## Background

The SARS-CoV-2 pandemic has resulted in millions of infections and deaths across the world. South Africa has the highest infection rate in Africa [1]. However, the number of COVID-19 deaths may be underestimated, based on excess death numbers recorded by the South African Medical Research Council [2]. The initial absence of effective treatments or vaccines resulted in drastic public health measures, including national lockdowns, social distancing and contact tracing, to reduce transmission [3]. These measures caused significant socio-economic disruption, particularly on the African continent [4]. The COVID-19 pandemic also disproportionately affected a broad range of vulnerable populations and compounded existing health inequities [5]. In South Africa, the COVID-19 pandemic was also associated with increases in mental health issues, gender-based violence, and substance abuse [2]. There was also limited access to health care, particularly for antenatal and postnatal services, HIV and TB testing and treatment, and non-communicable diseases such as cancer [6].

Research is a critical aspect of the response to public health emergencies. Research groups globally have focussed research efforts on COVID-19 disease pathogenesis and management strategies, including therapeutics and vaccines, and numerous clinical trials have been conducted [7]. While clinical research trials during COVID-19 have predominantly focussed on vaccine trials and drug development or repurposing, such as Chloroquine and Ivermectin, the African Academy of Sciences recommended that research to inform preventative and primary healthcare measures should form an essential component of Africa’s COVID-19 research agenda [8].

In the early phases of the pandemic, it was proposed that the urgency to produce effective COVID-19 therapeutics and vaccines warranted the consideration of potential modifications to the current elements of the research process. Some suggested modifications included omitting animal studies before Phase I trials in humans, or omitting Phase II trials [9, 10]. Previous disease outbreaks and public health emergencies [11–14] have also highlighted the challenges of balancing the need to uphold research ethics principles and standards with the need to produce valuable knowledge quickly [15]. Several international guidelines have also emphasised the importance of maintaining ethics standards for ethical research during public health emergencies and disease outbreaks [16–20] and the requirement for rapid, robust research ethics review during public health emergencies [21–23].

However, despite the availability of international guidelines to facilitate ethical review and oversight of research during the COVID-19 pandemic [24, 25], there is limited understanding of the structural, social, and contextual factors that may have impacted on the preparedness of research ethics committees (RECs) in Africa to operationalise or implement these guidelines, especially during the height of the pandemic. In addition, RECs in South Africa also needed to consider issues such as historical mistrust of research seen in social media, with hashtags such as #AfricansAreNotGuineaPigs, and to ensure equitable access to effective treatments or vaccines for COVID-19 [4].

In South Africa, the National Health Act 61 of 2003 (NHA) provides statutory authority for governance of health research and the necessary research ethics regulatory infrastructure [26, 27]. The National Health Research Ethics Council (NHREC) was established in 2006 in terms of Section 72 of the NHA [27]. The main functions of the NHREC include setting norms and standards for health research and clinical trials in South Africa, to determine guidelines to facilitate best practice for RECs, and to provide oversight for RECs in South Africa [26]. Section 73 of the NHA requires every institution, health agency, and health establishment at which health research is conducted, to establish or have access to a REC that is registered with the NHREC. Only NHREC-registered RECs may review and approve health research in South Africa (NHA s 71(1)(a) read with s 73(2)) [26, 27]. In South Africa, there are currently 46 RECs that review health research involving human participants registered with the NHREC [28].

The composition of RECs is outlined in Section 4.4 of the National Department of Health’s Ethics in Research Guidelines (2015), which emphasise that RECs should be independent, multi-disciplinary, multi-sectoral, and pluralistic. Research ethics committees are required to comprise a minimum of nine members, with representation from as many disciplines, sectors, and professions as possible, appropriate to the remit of the particular REC. The composition of RECs must include ethnically and culturally diverse members; an appropriate mix of males and females; lay persons, preferably from communities in which research is conducted; researchers who do not do human participant research; and members from other disciplines [26]. REC members are also required to undergo research ethics training at least once every three years [26].

The South African research ethics landscape was complicated by the absence of the NHREC for a significant duration of the COVID-19 pandemic. The NHREC was not re-constituted, without explanation, from November 2019 to December 2020 [29, 30]. Therefore, South African RECs needed to independently navigate their way through the first and second waves of the COVID-19 pandemic without national research ethics guidance and oversight. It is also unclear how research ethics oversight was adapted to different emergency contexts across the country or how RECs might have differently interpreted or implemented the National Department of Health’s Ethics in Research (2015) guidelines for research during public health emergencies [26] or international guidelines for research during the COVID-19 pandemic [24, 25]. Our research aimed to explore the perspectives and experiences of South African RECs regarding the ethical challenges of COVID-19 research in South Africa.

## Methods

The study was designed as a qualitative exploration of pertinent ethical issues that arose during REC deliberations of COVID-19 related research. We conducted in-depth interviews with REC chairs and members from large health science institutions across South Africa.

### Sample

We used a purposive sampling technique to identify REC chairs or members from health science institutions across South Africa. Potential participants were also identified through snowball sampling. Research ethics committee chairs and members of RECs registered with the NHREC from universities who formally train students in health science professions and conduct health research were eligible for inclusion in this study. Participants were required to have been actively involved in rapid review of COVID-19 research, including all levels of research risk, to be included in the study. We purposively identified REC chairs and members who were involved in the review of COVID-19 clinical trials, including preventative research, therapeutic trials, and vaccine studies. Participants were also required to have regularly attended REC meetings within the six-month period prior to study commencement. Potential participants were sent invitations to take part in the study via email. Diversity of representation of REC chairs and members from different health institutions across South Africa was attempted.

We contacted 31 REC chairs and members and 21 (67.7% acceptance rate) accepted an interview. The participants were based at seven universities across South Africa and all participants served as Chair or member of their institutional REC. In addition, two participants also served on two different national governmental organisation RECs, and a further two participants were also members of the REC for a national charitable foundation. Therefore, 10 RECs were represented in this study, which comprised 21.7% of all RECs (n = 46) registered with the NHREC. It is also estimated that the RECs represented in this study were responsible for over 75% of all moderate- to high-risk COVID-19 research, including candidate therapeutics research and candidate vaccine research conducted in South Africa during the study period (NHREC, personal communication, 29 November 2021).

We did not collect personal identifying information from participants to help protect confidentiality and to avoid identifying research ethics committees and institutions. Eleven participants had formal postgraduate training in bioethics, applied ethics, and research ethics. All participants had completed basic research ethics training within the three years prior to study participation. Research ethics committee chairs and members health-related qualifications and training included social science (n = 2), laboratory and medical science (n = 4), clinical specialists (n = 5), health professionals (n = 6), and members trained in professional care (for example, social work) (n = 2). The descriptive characteristics of participants are shown in Table 1.

**Table 1:** Summary of descriptive characteristics of participants (n = 21)

Participant code	Gender	REC status	REC experience (years)	Ethics qualificationa	Expertise
REC1	Male	Chair	> 15	Postgraduate Diploma	Clinical specialist Regulatory affairs Research ethics
REC2	Female	Chair	10–15	Masters	Health professional Bioethics
REC3	Female	Member	10–15	Postgraduate Diploma	Professional care Research ethics
REC4	Female	Chair	> 15	Honours	Health professional Qualitative methods Applied ethics
REC5	Male	Chair	> 15	–	Medical scientist Quantitative methods
REC6	Female	Member	10–15	Postgraduate Diploma	Health professional Research ethics
REC7	Female	Member	1–5	–	Laboratory scientist
REC8	Female	Member	> 15	Doctorate	Bioethics Social scientist
REC9	Male	Member	6–10	–	Clinical specialist Clinician researcher
REC10	Female	Member	1–5	–	Health professional
REC11	Female	Member	10–15	–	Clinical specialist Quantitative methods
REC12	Female	Member	10–15	–	Health professional Qualitative methods
REC13	Female	Member	1–5	–	Clinical specialist
REC14	Male	Chair	6–10	Postgraduate Diploma	Laboratory scientist Research ethics
REC15	Female	Chair	> 15	Doctorate	Clinical specialist Research ethics Biostatistics
REC16	Female	Chair	> 15	Postgraduate Diploma	Health professional Research ethics
REC17	Male	Chair	> 15	–	Professional care
REC18	Female	Chair	6–10	Postgraduate Diploma	Health professional Research ethics
REC19	Female	Member	1–5	–	Social scientist
REC20	Female	Chair	10–15	–	Laboratory scientist
REC21	Female	Member	> 15	Doctorate	Legally qualified Research ethics

^a^denotes formal ethics qualification. All participants had undergone research ethics training in the last three years

### Data collection

A clinical health professional trained in bioethics and qualitative research methods (TB) conducted the in-depth interviews with REC chairs and members. Her previous experience as a chair and deputy chair of two RECs in South Africa provided a unique opportunity to identify highly experienced REC chairs or members, many of whom have national or international profiles in research ethics and collect meaningful data.

Interviews were conducted remotely and online to maintain social distancing and reduce any potential infection risks during the COVID-19 pandemic. Interviews took place from January 2021 to March 2021 and lasted between 60 and 125 min each. Participants were asked to choose a private, convenient location for the interviews that protected the confidentiality of information shared during their interview. Interviews were conducted using Zoom, which routinely records both video and audio data. Therefore, video-recordings were deleted immediately after the interview to avoid storage of video material that could identify participants. Participants were also verbally reminded of these recording and storage arrangements prior to the commencement of the interviews. No other personal identifying information was captured. Interviews were conducted in English using an in-depth interview guide. The interview guide included questions regarding REC meetings, standard operating procedures and review processes during COVID-19, reciprocal review and harmonisation of South African REC reviews, and ethical issues associated with COVID-19 research. Interviews were conducted until data saturation was achieved. Field notes were taken during the interviews. Transcripts were password-protected and coded and were stored in the study OneDrive folder. Audio-recordings and transcripts will be stored for up to five years and will be destroyed once all study findings are published.

### Analysis

Recordings of the in-depth interviews were transcribed verbatim and field notes were converted into data documents. One researcher (TB) reviewed the transcripts for accuracy and completeness by comparing the audio recordings with the transcripts. Two researchers (TB and a research assistant) developed the codebook by independently coding four transcribed interviews. Line-by-line coding of transcripts was performed, and data were organised into themes and sub-themes. To improve inter-coder reliability, the coding of these interview transcripts and emerging themes were collectively discussed with the full study team (TB, SR, KM).

The coding was refined until an acceptable level of consensus and inter-coder reliability was achieved. Thereafter, two researchers coded all transcripts (TB and a research assistant). As interviews were coded, the codebook was expanded as required to ensure that novel responses were captured for analysis. Previously coded transcripts were checked to code any novel responses that had not been previously captured. An inductive approach to thematic analysis was used to analyse data. Atlas.ti (Version 6.2.28 Windows, ATLAS.ti Scientific Software Development GmbH, Berlin, Germany) was used to facilitate data analysis. During analysis, the full study team (TB, SR, KM) consulted regularly to review interpretations and discuss results.

### Ethics

This study was approved by the Health Research Ethics Committee at Stellenbosch University (N20/10/062_COVID-19) and the Faculty of Health Sciences Human Research Ethics Committee at the University of Cape Town (HREC REF 045/2021) and was guided by the Declaration of Helsinki’s ethical principle [31]. In addition, permissions from respective institutional gatekeepers were obtained to access potential participants from different institutions as required. All participants signed a consent form stating that they understood the nature and purpose of the research and that they agreed to their interview being recorded. All personal and institutional identifying data were removed from the interview transcripts before coding and analysis.

## Results

Our qualitative analysis revealed five main themes related to key ethical issues that arose during REC deliberations of COVID-19 related research. The themes and sub-themes are outlined in Table 2.

**Table 2:** Five main themes and related sub-themes identified during qualitative analysis

Themes	Sub-themes
Rapidly evolving research ethics landscape	Risk to benefit analysis as a moving target Placebo use in ongoing vaccine research Standard of care Post-trial access Social value
Extreme vulnerability of research participants	Individual and population vulnerability COVID-19 stigmatisation and prejudice Therapeutic misconception
Unique challenges to informed consent	Impact of fear and isolation on individual autonomy Challenges ‘on the ground’ Divergent views on consent waivers
Challenges to community engagement during COVID-19	RECs own role in community engagement The ‘who, when and how’ of community engagement during a pandemic
Overlapping research ethics and public health equity issues	Inequitable burden of research Equity in research agendas Inequities in access

### Rapidly evolving research ethics landscape

We asked participants to discuss common ethical issues identified in their REC’s discussions of COVID-19 related research. A central theme throughout the interviews was that the research ethics landscape is rapidly evolving during COVID-19 (see Table 3). Research ethics committee chairs and members were highly cognisant of the speed at which COVID-19 evidence is emerging and the associated impact on REC decisions. The transition from ‘is there equipoise’ and the REC’s approval of research to ‘evidence of harm’ and the REC’s need to withdraw approval or place approved studies on hold was seen as a reflection of the challenges to RECs and was reported consistently by REC chairs and members across different institutions. Risk to benefit analysis was seen as a moving target, with changes in excess death rates, pandemic waves, rapidly emerging evidence, and national vaccine rollout plans all intersecting and making deliberations complex.

**Table 3:** Quotations for the theme of ‘Rapidly evolving research ethics landscape’

Sub-themes	Quotations
Risk to benefit analysis as a moving target	What precedents can we live with? (REC3) I think the vaccines, the new vaccines, directed vaccines, repurposed vaccines, I think the risks have not all been elucidated because they bought them on out quite quickly as best as you can. And so, we have to deal with different decision making around not understanding all the risk and accepting the fact that there may be adverse effects that haven’t been identified, but getting the vaccine out there with proper reporting, proper monitoring, evaluation, and learning from overseas (REC1) And many of the other repurposed medicines or the medicines that are being used now, the preclinical work has not been to the level of we usually accept… so we would want to make sure that we’ve adjudicated appropriately on the risks (REC15) …the fact that we have a global emergency, doesn't mean we should throw all research and scientific standards overboard and just dive into anything and everything and hope that there will be an outcome that we like, and that that's probably something that sometimes gets a little lost, if you've got one trial after another coming through one protocol and you are seeing the patients dying in the hospital (REC8) …it's difficult because you've got the tension, you don't want to be too restrictive, because, you want obviously wanting research to progress, but it's a challenging space (REC19) …there are potential justice issues there. But I don't know how you balance justice with harms, potential harms and safety. And you have to call it somewhere. I suspect patient safety would be a little bit higher on the hierarchy than justice. But yeah, I think there are some areas that might be seen as being unjust, but I think there are usually good reasons for it (REC9)
Placebo use in ongoing vaccine research	…the biggest conversation is when we stop and decide especially from a placebo point of view, when a placebo is not important (REC20) …how can you allow people in studies to carry on not being vaccinated and following them up for the next few months, knowing that they could have been protected by the vaccination and after all, a trial is supposed to be a high resource environment, so they're supposed to be able to afford these sorts of things? (REC8) Of course, now, of course, the latest issues with the potential lack of efficacy with “South African variant” does that now open the door to placebo-controlled trials again, but I mean, still you don't know (REC11)
Standard of care	Things were viewed more in terms of a public health emergency than in terms of just pure science as they normally are…in the age of COVID, we've kind of viewed it a little bit differently. Perhaps what one might consider to be the best standard care has been assumed on the basis of what is being used, rather than what has been shown to be evidence based. Usually, we would want the best standard of care in the world to be on the basis of maybe two, phase three clinical trials or something like that what happens to the vaccine programme? What is that commentary when you can suddenly get vaccinated in emergencies in high-risk populations but, people are still running some clinical trials? (REC7)
Post-trial access	…how much post trial access became part of the deliberations in rapid views and trying to get our heads around protecting participants for putting themselves out there in an area that's so unknown, and what that means in terms of holding sponsors of especially the clinical trials accountable (REC2) Because people that have been on the vaccine trial, does that mean that their families should be eligible and get some sort of priority? I don't know. And it became quite a fraught issue, but the positive of it is also conscientizing our reviewers to looking at issues of post-trial access (REC5)
Social value	Navigating those realities of for example the very ill patients in COVID-19… we had to carefully reflect on that and make plans around that. Because we also understand that this is important, this has to be done. For the greater good, it might not benefit that specific patient (REC4) …it didn't even have to be a guarantee, it just had to be a commitment that they would take steps to ensure that our people would in some way get access to vaccines. So there was goodwill, and all the rest of it. But that's an ethical issue, our people put their necks on the line and have contributed to the success of that vaccine, which, after all, is going to make them an awful lot of money. And we are just sort of left carrying the can. I am not saying that we won't benefit eventually. But there was no commitment upfront, no stipulation that we would have any kind of benefits from, from allowing our people to participate in those trials (REC6)

In addition, the RECs ability to evaluate the robustness of evidence and how to incorporate non-peer reviewed publications and preprint manuscripts in deliberations of risk to benefit ratio and equipoise was a specific ethical concern. Many participants also identified ethical issues associated with the review of complex adaptive trial designs, standard of care, placebo use in vaccine studies, post-trial access, and benefit sharing. Potential social value and harms of COVID-19 related research were also a common ethical concern. Research ethics committee chairs and members were cognisant of a paradigm shift towards an increased focus on public health ethics in REC deliberations. Ethical issues associated with implementation trials during the COVID-19 pandemic were also highlighted.

### Extreme vulnerability of research participants

The majority of participants highlighted the extreme vulnerability of individuals and communities during the pandemic broadly, and during COVID-19 related research more specifically (see Table 4). Stigmatisation and prejudice were commonly identified social harms. The significant potential for therapeutic misconception associated with research was highlighted. Many participants commented that the fear of severe illness or death from COVID-19, particularly before effective vaccines were identified, increased the risk of therapeutic misconception. They also questioned the adequacy of informed consent processes to mitigate therapeutic misconception. Some participants were of the view that the urgency at the height of the pandemic rendered traditional considerations of therapeutic misconception irrelevant. This was countered by other participants who felt that a more rigorous approach was needed to mitigate therapeutic misconception due to significant fear, anxiety, and desperation that could unduly influence decisions to take part in research during the COVID-19 pandemic.

**Table 4:** Quotations for the theme of ‘Extreme vulnerability of research participants’

Sub-themes	Quotations
Individual and population vulnerability	…this is a vulnerable group of people who are desperate for treatment for prevention, for cure, and for getting lives back into some semblance of normality. And so there’s a desperation amongst the population and as such, that that heightens the vulnerability (REC7) …generally the population at the moment is vulnerable, regardless of whether there’s a formal vulnerability attached, people are scared, people are anxious, people are out of work, you know, there’s all these pressures attached. And so, I think there’s huge community vulnerability at the moment (REC5) …the participants are very vulnerable, no family around, often died without family or otherwise, were singled out of any contact whatsoever. And you may find that a lot of the clinical staff were putting barriers in front of them as well. And that’s obviously terrible (REC1) …for anybody coming into hospital with COVID infection means that, you know, you’re really sick, and you might die. And regardless of how hypoxic you are at that point, there is that fear, that vulnerability (REC9)
COVID-19 stigmatisation and prejudice	…being COVID-19 positive and the ospitalizedn and prejudice that has arisen due to this being a notifiable disorder and the fear of the disorder (REC17) There’s massive ospitalizedn in the community. Families, very similar to HIV, families who had a particular patient with COVID-19 are deemed to be a massive threat to the community (REC1)
Therapeutic misconception	I think therapeutic misconception is very high. I think the participants are very vulnerable, and I don’t think we understand how vulnerable participants are (REC2) A big misconception I guarantee you has occurred, I think people just volunteered because once you know how dangerous this disorder is, people may or may not have given voluntary, informed consent, not that they were coerced (REC13)

### Unique challenges to informed consent

Informed consent was identified as a key ethical challenge in the review of COVID-19 related research. Issues included procedural and pragmatic aspects of obtaining socially distanced informed consent as outlined in Table 5. Research ethics committee chairs and members highlighted the importance of understanding the reality of the clinical environment during a pandemic and what was feasibly possible in terms of socially distanced consent processes. Participants highlighted the potential effects of fear associated with COVID-19 infection, desperation for an effective vaccination or cure, and isolation from partners or family members on the validity of informed consent. Research ethics committee chairs and members also cited complexities associated with delayed consent processes and consent waivers for COVID-19 research. Participants also had divergent views on when consent waivers or delayed consent were permissible.

**Table 5:** Quotations for the theme of ‘Unique challenges to informed consent’

Sub-themes	Quotations
Impact of fear and isolation on individual autonomy	Even in terms of proxy consent, where families are not allowed to accompany patients at the moment or to visit…with layers of grief, and I suppose it’s exacerbated with anxiety from COVID is that families don’t fully understand or comprehend the information (REC6) There are so many issues with informed consent. And I do have to wonder if we are respecting autonomy, if we are ticking boxes a lot of the time (REC8) It’s a broader issue, not just related to the pandemic, but at the moment, I’m really worried about poor Mr. Jones getting his 10 different documents to sign. And, you know, and, and he’s doing it in isolation, by himself, he is scared in hospital, his family aren’t with him, you know, what choice does he really have? (REC11)
Challenges ‘on the ground’	Informed consent in a pandemic is such a minefield to navigate …it’s really ill patients that are hospitalized and there are issues of capacity to consent, who takes consent, the pragmatic realities around consenting (REC3) Again, it’s this balance between ensuring autonomy and overburdening and duplication and safety to the researcher (REC12)
Divergent views on consent waivers	We’re in a crisis situation in terms of ability to get next of kin aspects where we thought that independent witnesses would be freely available and be devised appropriately… (REC21) I think we just need to work on in terms of when is the right time to speak to the family. What is the information and how should we be transferring information to family members? (REC4)

### Complexities of the ‘who, when, and how’ of stakeholder engagement

Interestingly, participants identified two sub-themes related to stakeholder engagement presented in Table 6 below. The first sub-theme was the RECs own role in stakeholder engagement. Many participants equated this to the requirement for community or lay representation in the composition of RECs, as outlined in the South African Department of Health’s Ethics in Research Guidelines (2015). The contribution of lay members to REC deliberations was generally viewed as limited or unsatisfactory. Several recommendations for improved community representation and participation in REC deliberations were made.

**Table 6:** Quotations for the theme of ‘Complexities of the ‘who, when and how’ of stakeholder engagement’

Sub-themes	Quotations
RECs own role in stakeholder engagement	We struggle to get authentic, layperson representation on the committee. It’s hard for lay people to have a voice on a professional committee (REC20) So, I think we’ve missed a trick there, in terms of actually talking to Mr and Mrs community… this institution hasn’t gauged the community’s understanding about COVID-19 and where things are and I think a lot of that could have been done, stepped up quite quickly (REC11) Who’s the community member, then I wonder, you know, who does this person represent? For years? So, I think that’s a key concern for me is, who they are, and how we actually appoint them, which of course, isn’t clear… And also, then within the time of COVID, it is very difficult to now suddenly get input from other community members (REC18) We miss an opportunity with the role of ethics committees in public education. Our communities, some of them tend to see doctors as next to God… And then when you have a doctor that says, we’ve skipped stages in research, I tell you, I held my head and I just thought, this is exactly what we don’t need (REC3)
Challenges to community engagement during COVID-19	What do you do with community engagement in in pandemic times or in emergencies? None of our researchers used existing structures like they were doing for TB research or HIV/AIDS research (REC16) Are we getting true community sentiment? Or is that voice already too schooled in research to actually give you a true parameter of what's happening at community level? (REC3) As far as COVID is concerned, I really don't know who you consider to be the community… Who actually do you go to when you've had 1.4 million cases of COVID in the country, you can't just choose seven people off the street and say, that's the community? (REC7) You’ve got to have a large number of people to truly call that community… I don’t think it’s something that can just be a box that you tick, you’ve got to take it seriously… So, community involvement should be a whole lot of checks and balances and should be broader in the context of COVID specifically, in my opinion. If you want it to be truly valid (REC10)

The second sub-theme related to community or stakeholder engagement in research trial processes. Participants expressed that the urgency around research ethics review and approval of COVID-19 related research limited the potential for rigorous or authentic community/stakeholder engagement. The challenges associated with being able to delineate who represented a community and how effective community engagement could occur during COVID-19 were recognised.

### Overlapping research ethics and public health equity issues

Research ethics committee chairs and members identified that, during the COVID-19 pandemic, there seemed to be a significant overlap between research ethics and public health equity issues (see Table 7). A consistent example that was shared was the prioritisation of COVID-19 related research during the national lockdown and the halting of non-COVID related research, including therapeutic clinical trials, HIV and TB research, except for essential research visits that directly benefitted participants.

**Table 7:** Quotations for the theme of ‘Overlapping research ethics and health equity issues’

Sub-themes	Quotations
Inequitable burden of research	…most of the research has happened in in the public sector if we are honest with each other. And is that fair ethically in terms of the burden of research, and therefore, what is the right of that participant or that grouping, that community post-trial? (REC3) I've seen where we could… have been participant advocates in this process (REC14)
Equity in research agendas	From an equity point of view within the research environment, I think that investigators who have big organisations that can absorb this slowness, were able to move forward and to utilise this opportunity to find a niche to find spaces, I think that smaller units would have struggled and, and especially postgraduates would have struggled (REC1) We tried to be equitable. But we had to make very hard decisions around the research agenda within the institution (REC17)
Inequities in access	From an equitable point of view, despite the fact we in a pandemic, there are a lot of gaps in research in other vulnerable populations (REC4) You could suggest that that in some of the prevention studies, some of the participants that weren't being researched, directly, may have been able to benefit, because they're high risk and may have been absorbed into prevention studies… it's hard to know if that occurred. But equity got lost a little bit, I think in this pandemic (REC15)

Similarly, the prioritisation of clinical research that held therapeutic benefit during the recommencement of research during the COVID-19 pandemic also raised equity concerns. Critical social sciences and educational research was not prioritised, and many participants expressed awareness that this may have contributed to increased health-related inequities and structural social harms.

Implementation trials also raised health equity concerns among participants. Research ethics committee chairs and members were specifically challenged by emerging ethical issues related to vaccine access through participation in implementation research and the blurring of lines between research and vaccine prioritisation.

## Discussion

Public health emergencies such as the COVID-19 pandemic pose numerous challenges to health-related research [32]. The morbidity and mortality rates associated with COVID-19 infection and the social and economic burdens of government measures to limit infection spread contribute to widespread disruptions, distress, and uncertainties associated with the pandemic [33]. In the African context, research also often takes place in the context of historical inequities and ongoing power imbalances. People who are most disadvantaged or vulnerable, through poverty, marginalisation, or lack of access to healthcare are often disproportionately affected [4, 32, 34].

Emmanuel et al. [35] identified principles and benchmarks that are necessary for ethical conduct of research. These principles and benchmarks were developed to provide uniform and consistent ethical guidance and to minimise the possibilities of exploitation during multinational research. The ethical principles include collaborative partnership, social value, scientific validity, fair selection of study population, favourable risk to benefit ratio, independent review, informed consent, and respect of recruited study participants and the study population [35]. In our study, RECs consistently applied these principles in their deliberations of ethical issues in COVID-19 research. These ethical principles and benchmarks have also been identified in other studies investigating ethical challenges in pandemic or public health emergency research [36–44].

However, in our study, RECs were tested in their deliberations around the intersections of research ethics, public health ethics, and global health ethics that were presented by the COVID-19 pandemic and conducting research in emergency settings with national lockdowns. For example, the halting of non-COVID related research by government order raised significant public health equity issues. The stopping of non-COVID related research included therapeutic clinical trials, HIV and TB research, except for essential research visits that directly benefitted participants. South African RECs were mandated to facilitate a responsible and ethical approach to research involving human participants in the context of COVID-19. Limiting infections, preventing transmission, and protecting research participants, their communities, and research staff during the pandemic and national lockdowns were the primary considerations. Research ethics committees were also challenged to adapt quickly to changes in roll-out of the different stages of the South African Government’s COVID-19 Risk Adjustment Strategy. This was also compounded by the absence of the NHREC from November 2019 to December 2020 and resulted in RECs being left without formal national-level guidance and support from the NHREC during the critical first and second waves of the COVID-19 pandemic [29, 30]. In addition, when non-COVID related research was permitted to resume, social sciences and educational research was not prioritised, and many participants expressed awareness that this may have contributed to increased health-related inequities and structural social harms.

It has also been recognised that the initial response that was focussed on reducing the COVID-19 death toll and limiting COVID-19 infections had a devastating collateral effect on health and research equity [5]. Social isolation and movement restriction negatively impacted on health, but also limited access to basic health needs [45, 46]. This was compounded in the South African context, where halting of research restricted access to important ancillary health services for participants enrolled in clinical trials. In addition, with limited health and research resources, the diversion of funds and human resources to the fight against COVID-19 has resulted in disproportionate neglect of other infectious diseases such as HIV and TB [47, 48]. These inequities point to the need for a careful and balanced approach that considers human rights protection, public health concerns, research ethics, and social measures to avoid discrimination against vulnerable populations during public health emergencies [5]. Further, RECs struggled with balancing the evaluation of risk to benefit ratios for individual participants with the urgency associated with public health emergency research, and within the broader context of global inequities in vaccine access. Rid et al. [49] emphasised that while traditional ethical principles that guide clinical research should be retained as a reference point for ethical conduct of pandemic research, accelerating the process of delivering safe and effective treatments and vaccines against COVID-19 is a moral imperative [49]. However, ethical considerations should not be confined to one-off processes of review and REC deliberations should not occur in a vacuum [32]. Wright [32] explained how research ethics issues arise through the lifecycle of research and develop from initial setting of research priorities and funding to the translation of findings into clinical practice or public health implementation. Research ethics issues are also the responsibility of multiple stakeholders in the research process and are highly dependent on context [32]. It may therefore be appropriate to consider COVID-19 pandemic research partly through a public health lens.

Willison et al. [50] developed a framework and process to guide ethical reflection on public health projects through their lifecycle, from initial planning to knowledge exchange. These include relational autonomy (individual within community), social justice, reciprocity, respect for recruited participants and communities, and concern for welfare (favourable risk to benefit ratio). This framework retains core research ethics principles of respect for persons, concern for welfare, and justice but is augmented with concepts of relational autonomy and respect for communities, the inter-relationship of individual and community welfare, solidarity, and the common good. In particular, the positive obligation to promote social justice and the importance of reciprocity in public health emergency research, when individual participants carry risk or substantial burden for the benefit of others, are emphasised in this framework [50].

Further, the Nuffield Council on Bioethics have proposed an ‘ethical compass’ of three core values, namely equal respect, fairness, and helping reduce suffering to support ethical reflection for global health research at the level of policy, as well as on the ground. They also suggest that research funders, institutions, governments, and research journals have a duty to ensure that the research they fund, support, or publish is compatible with these three core values [32]. This also broadens the responsibility and accountability for the ethical conduct of research during the COVID-19 pandemic.

However, despite the availability of international guidelines for research during public health emergencies [18, 51–54], in the South African context, there was a lack of clear national guidance to operationalise important ethical considerations for research during COVID-19. For example, the absence of clear guidance to operationalise mutual recognition of ethics review resulted in RECs being underprepared to reduce duplication of effort in urgent research and in conducting rapid reviews of COVID-19 research. In addition, differences in interpretation of national guidelines regarding appropriate limitations of public health emergency research and informed consent processes by individual RECs resulted in disparate ethical reviews and differences in informed consent practices across study sites [29, 55]. This was also compounded by the absence of the NHREC during the height of the COVID-19 pandemic, which prevented clear national ethical leadership and updated national guidelines to facilitate ethical review of COVID-19 research.

In South Africa, the absence of the NHREC also resulted in the development of a collaborative network of research ethicists during the first wave of the COVID-19 pandemic. This network was called Research Ethics Support in COVID-19 Pandemic (RESCOP) and served to provide a repository of ethics resources for public health emergencies and COVID-19, to facilitate collaboration between individual RECs in the absence of the NHREC and to develop guidance to operationalise ethics review of COVID-19 research. The RESCOP network also developed guidelines for rapid full committee review of COVID-19 treatment and prevention trials [29]. These guidelines were developed to align with, and supplement, the National Department of Health’s Ethics in Research Guidelines (2015) [26] and were compliant with the subsequent WHO guidance for rapid review of COVID-19 research [54]. The RESCOP rapid review guidelines emphasised the high vulnerability of research participants during the COVID-19 pandemic but cautioned RECs against being overly restrictive and advised that ethical approval processes should occur very rapidly but without compromising the rigour of ethics review. However, while rapid review processes for individual RECs were implemented effectively, efforts to harmonise research ethics review and implement reciprocal review did not work well. This highlighted the need for clear national research ethics guidance to facilitate effective reciprocal review processes, and to ensure the preparedness of RECs for future public health emergencies [29].

In our study, concerns regarding informed consent processes were one of the main ethical considerations identified by RECs in their evaluation of COVID-19 research. Consent issues included procedural and pragmatic aspects of obtaining socially distanced informed consent, the realities of obtaining informed consent on the ground in challenging and often fraught clinical environments, and what was feasibly possible in terms of socially distanced consent processes. Factors impacting on the voluntariness and validity of informed consent processes, including fear associated with COVID-19 infection, desperation for an effective vaccination or cure, and isolation from partners or family members were consistently identified. Research ethics committees also cited complexities associated with delayed consent processes and consent waivers for COVID-19 research. These informed consent issues identified in our study resonate with other literature regarding issues with consent processed during the COVID-19 pandemic [27, 55–61]. Largent et al. [59] highlight how the COVID-19 pandemic has influenced practicability determinations of informed consent. They suggest that, as the pandemic continues, further guidance from bioethicists and regulators is needed to find the best way forward for informed consent approaches during COVID-19 [59].

Community engagement was another important ethical issue identified by South African RECs. In accordance with national research ethics guidelines, health researchers in South Africa are encouraged to engage stakeholders in their research [62]. However, community engagement has been complicated in the context of the COVID-19 pandemic, where decisions regarding lockdowns and physical distancing were made rapidly, with little or no time to engage local communities, especially around research. Saxena et al. [63] recommend increased community engagement in pre-pandemic or inter-pandemic times and consider that this approach might increase public trust [63]. Research ethics committees have a role to play in shaping researchers’ practices and involving local communities [62], and this is of particular importance in research during public health emergencies.

The COVID-19 pandemic has highlighted how uncertainty and risk of misinformation can influence community perceptions of research and vaccine hesitancy [64]. Community engagement is essential to build trust in research and show respect to communities [25, 34, 65]. This approach should also include good information management. However, models to practically implement authentic community engagement regarding COVID-19 research in the context of the pandemic are lacking. Further research is needed to explore RECs own role in community engagement and how to practically execute effective and appropriate community engagement during the COVID-19 pandemic.

Issues of equity and access were some of the critical ethical issues identified by RECs. As stated above, these debates extended beyond traditional standard of care debates within the South African context, and that RECs identified both public health and global health ethics issues that impacted on the conduct of COVID-19 vaccine trials in South Africa. A key issue was the use of placebo groups in vaccine trials in low- and middle-income countries (LMICs) that have limited or no vaccine access. A World Health Organisation expert group identified conditions for the ethical acceptability of a placebo group at the onset of a vaccine [66]. Some researchers have controversially suggested that there is an opportunity to continue to conduct randomised vaccine trials with a placebo group in LMICs where COVID-19 vaccine access is limited due to cost or a lack of infrastructure to store and distribute vaccines, as research trials will increase the overall number of vaccinated individuals [67, 68], although the percentage of national populations involved in research trials is likely to be very low. Beneficence and local stakeholder engagement are considered as important ethical principles for the ongoing use of placebo groups in LMICs [69]. However, considering the strong results for efficacy of several approved vaccines and participants’ contributions to research, the ongoing justification for placebo groups, even in LMICs, is limited [49].

### Strengths and limitations

While we were able to interview many of the research ethics leaders from seven of the large academic institutions across South Africa, the sample represents a small subset of REC members in South Africa. In addition, we only interviewed each participant once. Given the rapidly evolving nature of COVID-19 research ethics landscape, longitudinal data collection would have allowed us to explore emerging issues in more depth, and to understand changes in REC’s perceptions and experiences over time. However, despite these limitations to generalisability, our findings provide a strong base for future empirical research to further understand ethical challenges in COVID-19 research and their impact on REC deliberations. These findings can also be translated to inform and support the development of ethical policy and guidance for public health emergency research in the African context.

## Conclusion

Our findings provide unique insights into the perspectives and experiences of RECs regarding the ethical challenges of COVID-19 research in South Africa. Further comparative analysis between different countries is needed to develop the discourse around African RECs and COVID-19 research ethics issues. The numerous ethical issues identified also highlight the need for research ethics teaching and training, especially in informed consent, as well as the urgent requirement for the development of national guidelines for research ethics during public health emergencies.

## Data Availability

The datasets generated and analysed during the current study are not publicly available due to consent not being obtained for public sharing. However, data may be available from the corresponding author on reasonable request and with permission of the Stellenbosch University Health Research Ethics Committee and the University of Cape Town, Faculty of Health Sciences Human Research Ethics Committee.

## Abbreviations

REC(s): Research ethics committee(s)
NHREC: National Health Research Ethics Council
LMIC(s): Low- and middle-income countries
RESCOP: Research Ethics Support in COVID-19 Pandemic
